# An Efficient Method of *Pennisetum* × *advena* ‘Rubrum’ Plantlets Production Using the Temporary Immersion Bioreactor Systems and Agar Cultures

**DOI:** 10.3390/plants12071534

**Published:** 2023-04-02

**Authors:** Mariusz Pożoga, Dawid Olewnicki, Elżbieta Wójcik-Gront, Piotr Latocha

**Affiliations:** 1 Section of Horticultural Economics, Institute of Horticultural Sciences, Warsaw University of Life Sciences—SGGW, 02-787 Warsaw, Poland; 2 Department of Biometry, Institute of Agriculture, Warsaw University of Life Sciences—SGGW, 02-787 Warsaw, Poland; 3 Department of Environmental Protection and Dendrology, Institute of Horticultural Sciences, Warsaw University of Life Sciences—SGGW, 02-787 Warsaw, Poland

**Keywords:** in vitro, multiplication, ornamental plant, TIS, tissue culture

## Abstract

The aim of this study is to develop an efficient method for micropropagation of *Pennisetum* × *advena* ‘Rubrum’. Agar cultures containing Murashige and Skoog (MS) medium supplemented with 6-benzyl-amino-purine (BAP) in various concentrations (0.5 mg/L to 2 mg/L) and a temporary immersion bioreactor system (TIS) using liquid medium MS with an addition of 1 mg/L BAP were tested. Rooting was performed using ½ MS medium supplemented with different auxin combinations (indole-3-butyric acid IBA and α-naphthalene acetic acid NAA) and activated charcoal. The TIS method was found to be the most efficient, producing 36.9 new plants within four weeks. The resulting plantlets were thin and bright green in color, with no signs of hyperhydricity. The most suitable agar medium yielded 19.5 new plants within eight weeks. For rooting, ½ MS supplemented with 0.5 mg/L IBA and 0.5 mg/L NAA exhibited an 84% rooting rate, whereas the addition of activated charcoal inhibited rooting.

## 1. Introduction

*Pennisetum* is the genus within the Poaceae family that consists of over 80 species found across a wide range of climatic regions. The plant is relatively low-maintenance and drought-resistant but sensitive to low temperatures [1]. *Pennisetum* × *advena* ‘Rubrum’, also known as *Pennisetum setaceum* ‘Rubrum’ or purple fountain grass, is a highly desirable plant due to its growing popularity, beautiful appearance, ease of cultivation, and stress resistance [2]. *P.* × *advena* is considered to be a cross between *P. setaceum* from North Africa and *Pennisetum macrostachys* from Malaysia [3,4]. This plant is triploid (2 *n* = 3x = 27), rather sterile, and exhibits extremely low seed production, making in vitro techniques essential for large-scale propagation [5,6]. Previous research on *Pennisetum* in vitro propagation has mainly focused on somatic embryogenesis. Maity et al. [7], Lambé et al. [8], and Mythili et al. [9] presented somatic embryogenesis in *P. glaucum*, while Pius et al. [10] and Vasil and Vasil [11] studied somatic embryogenesis in *P. americanum*. Studies on *P.* × *advena* ‘Rubrum’ were mainly associated with obtaining new varieties [2]. Vegetative propagation techniques such as crown division and culm cuttings are alternative methods for propagating *P.* × *advena* ‘Rubrum’ [12,13]. However, these methods are dependent on the growing season, which is disadvantageous. In vitro techniques are more attractive for plant propagation because they are independent of seasonal and weather conditions. Since there is limited literature available on the in vitro propagation of *P.* × *advena* ‘Rubrum’ [14], further research is essential.

Increased competition in the plant market has prompted the exploration of more cost-effective methods of plant production. Bioreactors offer a potential solution. Different types of liquid medium bioreactors have been developed over the years, such as stirred tank bioreactors, cone balloon-type airlift bioreactors, rotating drum bioreactors, nutrient mist bioreactors, radial flow bioreactors, and wave bioreactors. However, the temporary immersion system (TIS), which includes the SETIS and RITA systems [15,16,17], has been the most widely used. TIS has many advantages, such as reducing the use of agar, which is the most expensive component of tissue culture media [18]. Additionally, liquid cultures provide more uniform culturing conditions by reducing medium exchange without container change or plant passage. Gas exchange, which speeds up growth, is also beneficial [19,20,21]. Despite these advantages, there are no reports on the micropropagation of the *Pennisetum* genus. There have been some reports on other Poaceae family members, such as *Arundo donax,* in TIS using the RITA system, which yielded 1200 plants in just six months from a single explant, which is about 100 times more than in conventional field propagation [22]. Da Silva et al. [23] conducted extensive research on TIS propagation in sugarcane, energy cane, *Miscanthus* spp., *Miscanthus sinensis*, *Erianthus* spp., S*accharum spontaneum*, and S*accharum* spp. × *Sorghum* spp. They reported good proliferation rates in the bioreactor system. TIS has been compared with agar cultures in several experiments, and TIS has been shown to have a significant advantage in proliferation. Alvard et al. [24] found that banana cultures in TIS yielded twice as much as those in agar. Gianguzzi et al. [25] also found that *Capparis spinosa* had a better growth rate, shoot length, and number of new shoots in TIS than in agar cultures. Similarly, Escalona et al. [26] found that *Ananas comosus* and Perez et al. [27] found that *Quercus suber* had a better proliferation rate in TIS than in agar cultures.

Immersion time is a crucial factor that affects the proper TIS operation. Teisson and Alvard [28] conducted research on *Coffea* sp. somatic embryos and used two immersion frequencies. They found that a 15-min immersion every 6 h induced the development and germination of embryos, while a 1-min immersion every 24 h on the same culture medium stopped embryo development. Etienne and Berthouly [19] observed that an increase in the frequency of short immersions (1 min) stimulated somatic embryo formation and improved its quality in *Coffea arabica* TIS. They found that for daily frequencies of 1, 2, and 6 immersions, yields of 480, 2090, and 3100 embryos were obtained, respectively. Research on immersion time and frequency can result in a significant increase in the development of new shoots or somatic embryos. Further research is needed to develop profitable commercial ornamental grass production, given the few reports on TIS propagation in the Poaceae family and the good results obtained in other species.

This study aimed to propose an effective micropropagation method for *P*. × *advena* ‘Rubrum’ and compare agar-based media with the TIS system to determine the most efficient micropropagation approach.

## 2. Results

**Figure 1:**
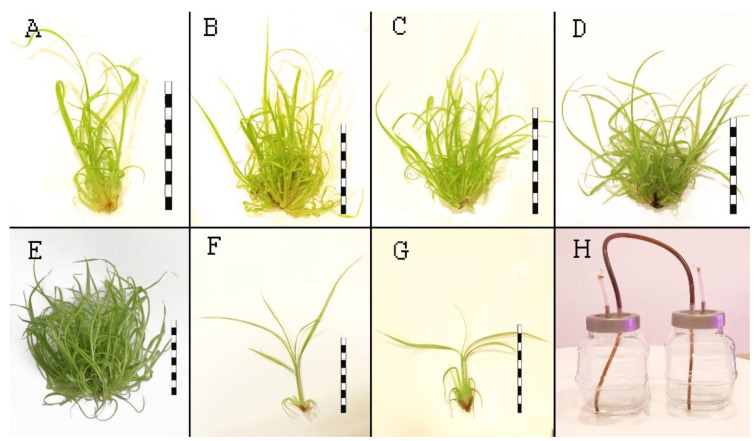
Effect of multiplication media. (**A**). Plants obtained after 8 weeks on M1. (**B**). Plants obtained after 8 weeks on M2. (**C**). Plants obtained after 8 weeks on M3. (**D**). Plants obtained after 8 weeks on M4. (**E**). Plants obtained after 4 weeks on M5 medium. (**F**). Representative single plant from M1, M2, M3, M5. (**G**). Representative single plant from M4. (**H**). Temporary immersion bioreactor system. Bars = 5.0 cm.

Among the agar media, M2 and M3 showed the best multiplication rate after eight weeks (Table 1, Figure 1B,C). In contrast, M1 and M4 resulted in significantly lower multiplication rates (Table 1, Figure 1A,D). Plants cultured in M1, M2, and M3 were thin and green in appearance (Figure 1F). In contrast, plants grown on M4 were very hard, slightly vitrified, and exhibited basal extension, with leaves showing slight reddening at the tip (Figure 1G). As M2 medium produced the best multiplication results, a concentration of 1 mg/L BAP was used for the TIS experiment. The highest number of 36.2 plants per explant was obtained in the M5 bioreactor, and this method produced significantly different results compared to the other methods. Means, medians, and minimum and maximum values are presented in Table 1. The morphology of the new plants was similar to that of plants cultured in M1–M3. TIS and plant images are presented in Figure 1E and Figure 1H, respectively. Based on the statistical analyses, it can be concluded that the sample distributions did not follow a normal distribution. Therefore, according to Kruskal-Wallis, the media differed significantly from each other (*p* < 0.05). Multiple comparisons of the samples revealed that the M1 and M4 media were not significantly different, and neither were the M2 and M3 media (Table 1).

**Figure 2:**
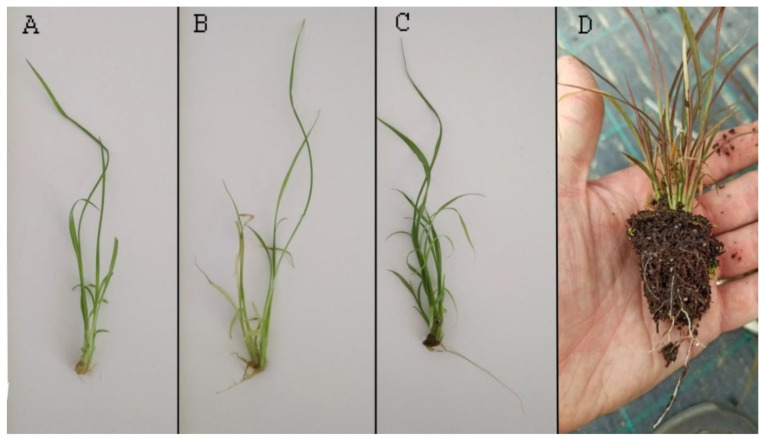
Effect of rooting medium and acclimatization. (**A**). Roots obtained on R1 after 3 weeks. (**B**). Roots obtained on R2 after 3 weeks. (**C**). Roots obtained on R3 after 3 weeks. (**D**). Acclimatized plant.

After three weeks, the rooting rate was determined. Among the tested media, R3 was found to be the most effective, with 84% of plants successfully rooted. The minimum number of rooted plants was five out of ten and the maximum was ten (Table 2). The roots were long, unbranched, and healthy (Figure 2C). Only 36% of the plants were rooted on R2 and just 12% on R1. The minimum number of rooted plants on R2 and R1 was two and zero, while the maximum number of rooted plants was six and three, respectively (Table 2). The roots that developed on R2 were shorter than on R3, and slightly branched (data not shown) (Figure 2B). The plants grown on R1 had very short, unbranched roots (Figure 2A). The media containing activated charcoal (RAC1, RAC2, and RAC3) strongly inhibited rhizogenesis and resulted in the complete absence of roots (data not shown). The rooted plants are shown in Figure 2D. Regarding the rooting results, the sample distributions did not match the characteristics of a normal distribution, similar to the proliferation results. Thus, according to Kruskal-Wallis, the methods differed significantly from each other (*p* < 0.05). Multiple sample comparisons indicated that all the methods were significantly different (Table 2).

In the next stage of the experiment, the rooted plantlets were transferred to multi-pots for acclimatization to greenhouse conditions. All plants successfully survived this stage (100% survival rate) and exhibited rapid growth.

## 3. Discussion

Efficient propagation methods are essential for ornamental plants with considerable market value, such as *P.* × *advena* ‘Rubrum’. In our study, the examined media had various effects and showed significant differences. The highest number of new plants was produced in the MS medium containing 1 mg/L BAP when using the common agar culture. Both higher and lower concentrations of BAP were found to inhibit plant propagation, a common observation also reported in other species such as the rose [29] or banana [30]. Wei et al. [14] identified MS with an addition of 1.5 mg/L BAP, 0.1 mg/L NAA, and 0.5 mg/L IBA as the most optimal medium for *P.* × *advena* ‘Rubrum’ micropropagation with a multiplication coefficient of 6.5 after 30 days. In our experiment on MS medium supplemented with 1.5 mg/L BAP without the addition of auxin, 18.1 new plants were obtained after eight weeks. These results suggest that passage time is also critical in *P.* × *advena* ‘Rubrum’ micropropagation in addition to medium composition. An elongated passage time on a similar medium ensured approximately a three-times better result. Very high levels of BAP in *Pennisetum* micropropagation can also generate a large number of new plants. MS with 4 mg/L of BAP allowed for the acquisition of 26.6 new shoots of *Pennisetum glaucum* after 30 days [7]. This author also used embryogenic callus regeneration and found that the results were dependent on both the explant type and genotype. The best results of 10.2 plants per callus were achieved after 30 days on MS with an addition of 2 mg/L BAP. Yue et al. [2] proposed a micropropagation method for triploid and hexaploid *P.* × *advena* using callus induction on MS supplemented with 3 mg/L 2.4-D, 1 mg/L NAA, and 1 mg/L KIN in the dark. The cultures were then transferred to MS medium containing 3 mg/L BAP and 0.5 mg/L NAA for 4–6 weeks for shoot induction before being transferred to ½ MS rooting medium. Other reports of micropropagation protocols for *Pennisetum* species via callus include the popular methods for *P. americanum* [10] and *P. glaucum* [8,9].

The highest rooting percentage of *P.* × *advena* ‘Rubrum’, reaching 84%, was observed in ½ MS medium supplemented with 0.5 mg/L IBA and 0.5 mg/L NAA. Wei et al. [14] achieved 100% rooted plants using the same medium, but differences could be due to the use of different genotypes. Activated charcoal is a commonly used substance in plant tissue culture due to its high adsorptive capacity. It is especially effective at adsorbing aromatic unsaturation products compared to olefinic ones. This makes it useful for adsorbing many auxins and cytokinins [31]. Activated charcoal can also accumulate inhibitory substances in the medium and phenolic compounds [32,33]. While many investigators have suggested that activated charcoal improves rooting [34,35,36], our study found that the addition of activated charcoal to any medium actually inhibited rooting, possibly due to the absorption of auxin by activated charcoal, as suggested by Fridborg and Eriksson [37]. A similar inhibitory effect on rooting was observed by Ben Joura [38] in the Dutch elm hybrid ‘Commelin’. Medium supplemented with 5 μM IBA and 2 g/L activated charcoal resulted in 46% rooting, while medium with the same IBA concentration but no activated charcoal resulted in 96% rooting. Additionally, the number of roots was higher when activated charcoal was absent. Buendía-González et al. [39] also found that the addition of activated charcoal to the induction medium instead of PVP led to a prolonged rooting time in *Prosopis laevigata* shoots, with root formation taking twice as long.

There are currently no reports available on the bioreactor propagation of *Pennisetum*. Temporary Immersion Bioreactors (TIS) appear to be the most promising systems for commercial tissue culture laboratories due to their compact size and ease of use. Moreover, TIS has the advantage of reducing hyperhydricity compared to permanent immersion [40]. In our experiment, we did not observe hyperhydricity and achieved 90% more new plants in TIS than in agar culture, and twice as quickly. Many researchers have also reported better performance of TIS over standard agar-based cultures. Comparing the same agar-based medium with TIS is a common research design to evaluate the efficiency of the two methods [41,42]. Murch et al. [20] demonstrated a five-fold bigger fresh weight per plantlet of *Crescentia cujete* and a two-fold better rooting rate. Businge et al. [43] observed an approximately 100% higher multiplication rate, 60% more fresh weight of *Betula pendula*, a 500% higher multiplication rate, and 1100% more fresh weight of *Eucalyptus* species. Uma et al. [44] found that the multiplication of new banana plant shoots in TIS was 2.7 times higher than in the semisolid culture method. Moreover, Yan et al. [45] and Jiménez et al. [46] observed higher multiplication of plants in TIS prior to agar cultures. It is important to note that TIS is usually used to determine the proper duration of immersion, intervals of immersion, or effects of explant density rather than plant growth regulator concentrations [19]. In our research, we used a 1min immersion time with a 1h interval to achieve short but frequent contact of the explant with the medium. Perez et al. [27] found that immersion for 1 min every 4 h induced 114 cotyledonary embryos in *Quercus suber*, which was significantly more than the 48 embryos obtained with immersion for 1 min every 6 h and the 14 embryos obtained with immersion for 1 min every 12 h. Similarly, Villegas-Sanchez et al. [47] demonstrated that more frequent immersion of *Rosmarinus officinalis* explants leads to excellent results. Immersion for 1 min every 12 h resulted in 170 new shoots, while immersion for 1 min every 24 h produced only three new shoots. Using immersion for 5 min every 12 h, 22 new shoots were obtained, and immersion for 5 min every 24 h resulted in only five new shoots. Based on this information, short but frequent immersion can yield good proliferation results.

The temporary immersion bioreactor method shows much higher efficiency than the agar cultures. Further investigation and method improvements should be developed.

## 4. Materials and Methods

### 4.1. Micropropagation Experiment Design

The *P.* × *advena* ‘Rubrum’ plant used in the experiment was acquired from a local nursery. The explant for culture initiation was a 1 cm intercalary meristem excised from mother plants. The explants were surface sterilized with 15% commercial bleach solution (4.28% sodium hypochlorite) for 15 min. Subsequently, they were rinsed with sterile distilled water three times, each for 5 min, 10 min, and 15 min. The culture initiation medium used was Murashige and Skoog MS [48] with vitamins, including 2 mg/L glycine, 100 mg/L *myo*-inositol, 0.5 mg/L nicotinic acid, 0.5 mg/L pyrodoxine, and 0.1 mg/L thiamine, and supplemented with 1 mg/L 6-benzyl-amino-purine (BAP). Different BAP concentrations in MS medium with vitamins were tested to identify the most efficient medium for multiplication, as shown in Table 3. The media used for rooting are also listed in Table 3. In addition to the bioreactor combination M5, every medium was supplemented with 2% sucrose and 7 g/L plant agar. The pH was adjusted to 5.8 before autoclaving.

The photoperiod consisted of 12 h of daylight and 12 h of darkness, with cool-white fluorescent tubes (3100 Lm) providing the light source at a constant temperature of 23 °C. The subculture duration was eight weeks for agar cultures and four weeks for TIS. Agar cultures were conducted in 350 mL plastic containers with ten explants each. The TIS bioreactor consists of two 1.8 L jars, with 400 mL of medium in the first jar and ten explants placed in the second jar. The immersion frequency was set to 1 min per hour. Observations were made after eight weeks for agar cultures, four weeks for TIS multiplication, and three weeks for rooting. The experiment was repeated three times, with ten containers containing ten explants each used for each treatment. The plants were acclimatized to greenhouse conditions in May, with humidity maintained at 80–90% for the first week and then gradually reduced by 10–15% per week. Shading was provided for the first three weeks.

### 4.2. Statistical Analysis

The statistical analysis of the experiment results was performed using STATISTICA version 13.0 software (TIBCO Software Inc. (2017), http://statistica.io. (Version 2017 installed on the disk), CA, Palo Alto, USA [49]). To determine whether or not the sample distribution followed a normal distribution, the Kolmogorov-Smirnov test was used. Since the data did not follow a normal distribution, the Kruskal-Wallis non-parametric ANOVA test was employed to evaluate the differences between the tested media. The Kruskal-Wallis test is a non-parametric statistical test used when data do not meet the assumptions of normality or equal variances required by the ANOVA. The test statistic is based on the differences between the mean ranks of the groups [50]. Dunn’s post-hoc test was used to determine which groups were significantly different from each other [51]. The level of statistical significance was set at *p* < 0.05 for all calculations.

## 5. Conclusions

In the micropropagation of *P.* × *advena* ‘Rubrum’, using a temporary immersion system with the addition of MS medium supplemented with 1 mg/L BAP resulted in 90% more new plants compared to agar cultures and in two times shorter time with no signs of hyperhydricity. This BAP concentration was chosen because it showed the highest multiplication rate in the agar medium. The TIS method produced statistically different results than the other methods. Rooting was achieved on ½ MS supplemented with 0.5 mg/L IBA and 0.5 mg/L NAA. The addition of activated charcoal inhibited rooting.

## Figures and Tables

**Table 1:** Statistical description (mean, maximum, minimum, Q1—the first quartile, median, Q3 —the third quartile, SD—standard deviation) of several newly emerged plants in used multiplication media.

Medium	Specification						
	Mean	Min.	Max.	Q1	Median	Q3	SD
M1	12.7 ^c^	4	22	8	12	17	5.2
M2	19.5 ^b^	7	34	13	20	26	7.1
M3	18.2 ^b^	5	31	12	19	25	7.3
M4	13.0 ^c^	3	24	8	12	18	5.7
M5	36.2 ^a^	15	55	31	36	41	7.7

Mean values marked with the different superscript letters differ significantly according to the Kruskal-Wallis post-hoc test: H (df = 4, *n* = 1500) = 785.20, *p* < 0.05.

**Table 2:** Descriptive statistics (percentage, mean, maximum, minimum, –1—the first quartile, median, Q3—the third quartile, SD standard deviation) of the rooted plants in each medium.

Medium		Specification						
	Percentage	Mean	Min.	Max.	Q1	Median	Q3	SD
R1	12	1.2 ^c^	0	3	0	1.0	2	0.9
R2	36	3.6 ^b^	2	6	3	3.0	5	1.2
R3	84	8.4 ^a^	5	10	8	8.5	9	1.2

The mean values marked with different superscript letters differ significantly according to the Kruskal-Wallis post-hoc test: H (df = 2, *n* = 90) = 75.22, *p* < 0.05.

**Table 3:** Media used in the agar and temporary immersion bioreactor experiments.

Medium	BAP mg/L	Agar	IBA mg/L	NAA mg/L	AC mg/L
M1	0.5	+	-	-	-
M2	1	+	-	-	-
M3	1.5	+	-	-	-
M4	2	+	-	-	-
M5	1	-	-	-	-
R1	-	+	-	-	-
RAC1	-	+	-	-	2
R2	-	+	0.5	-	-
RAC2	-	+	0.5	-	2
R3	-	+	0.5	0.5	-
RAC3	-	+	0.5	0.5	2

## Data Availability

All data is contained in the manuscript.
